# Microbial Community Structure and Function of Soil Following Ecosystem Conversion from Native Forests to Teak Plantation Forests

**DOI:** 10.3389/fmicb.2016.01976

**Published:** 2016-12-09

**Authors:** Vidya de Gannes, Isaac Bekele, Denny Dipchansingh, Mark N. Wuddivira, Sunshine De Cairies, Mattias Boman, William J. Hickey

**Affiliations:** ^1^Faculty of Food and Agriculture, The University of the West Indies at St. AugustineTrinidad and Tobago; ^2^Ministry of Agriculture, Land and Fisheries, The University of the West Indies at St. AugustineTrinidad and Tobago; ^3^O. N. Allen Laboratory for Soil Microbiology, Department Soil Science, University of Wisconsin-MadisonMadison, WI, USA

**Keywords:** forest, soil, bacteria, fungi, archaea, and Illumina

## Abstract

Soil microbial communities can form links between forest trees and functioning of forest soils, yet the impacts of converting diverse native forests to monoculture plantations on soil microbial communities are limited. This study tested the hypothesis that conversion from a diverse native to monoculture ecosystem would be paralleled by a reduction in the diversity of the soil microbial communities. Soils from Teak (*Tectona grandis*) plantations and adjacent native forest were examined at two locations in Trinidad. Microbial community structure was determined *via* Illumina sequencing of bacterial 16S rRNA genes and fungal internal transcribed spacer (ITS) regions, and by phospholipid fatty acid (PLFA) analysis. Functional characteristics of microbial communities were assessed by extracellular enzyme activity (EEA). Conversion to Teak plantation had no effect on species richness or evenness of bacterial or fungal communities, and no significant effect on EEA. However, multivariate analyses (nested and two-way crossed analysis of similarity) revealed significant effects (*p* < 0.05) of forest type (Teak vs. native) upon the composition of the microbial communities as reflected in all three assays of community structure. Univariate analysis of variance identified two bacterial phyla that were significantly more abundant in the native forest soils than in Teak soils (*Cyanobacteria, p* = 0.0180; *Nitrospirae, p* = 0.0100) and two more abundant in Teak soils than in native forest (candidate phyla TM7, *p* = 0.0004; WS6, *p* = 0.044). Abundance of an unidentified class of arbuscular mycorrhizal fungi (AMF) was significantly greater in Teak soils, notable because Teak is colonized by AMF rather than by ectomycorrihzal fungi that are symbionts of the native forest tree species. In conclusion, microbial diversity indices were not affected in the conversion of native forest to teak plantation, but examination of specific bacterial taxa showed that there were significant differences in community composition.

## Introduction

Soil microorganisms are an integral part of forest ecosystems as they play central roles in most nutrient transformations in forest soils. The stability and sustainable development of forest ecosystems rely on these nutrient transformations and the interactions between above- and below-ground components drives forest ecosystem processes (You et al., 2014). For the below ground processes, soil microbial communities are important in mediating soil organic matter decomposition, and nutrient cycling in these ecosystems (van der Heijden et al., 2008). The interactions between above- and below ground components of forest ecosystems have been receiving extensive research attention with a growing interest in ascertaining the effect of tree types on soil microbial community.

Forest trees influence the soil through many mechanisms including permeation of roots, input of organic matter through root litter and exudation of ions and organic compounds. Studies have reported that soil microbial composition and or community structure can be altered by plant species, plant diversity, vegetation or forest type through modifying the site microclimate and litter chemistry (De Deyn et al., 2008; Wardle et al., 2012; Jassey et al., 2013). For example, in a tropical montane forest, tree species effects on soil microbial community was investigated using microbial lipid analysis and it was reported that some tree species- microbial specificity was revealed between conifers and broadleaved species (Ushio et al., 2008). Using PCR-DGGE of rRNA, Shi et al. (2012) revealed significant differences in microbial communities *Alphaproteobacteria* and pseudomonads in genetically modified pine forests compared to native radiata pine. Even where there were conversions of tropical forest soils to agricultural (cultivated) soils, distinct microbial communities were revealed using phospholipid fatty acid (PLFA). Additionally, in four Alpine forest sites, shifts in bacterial and fungal richness and diversity at various altitudes were reported using a high throughput technique, Illumina sequencing (Siles and Margesin, 2016). But contradictory results were also reported by other researchers. For example, no significant differences were found in soil bacterial diversity, using pyrosequencing, along altitudinal gradients in forest soils (Shi et al., 2012; Wardle et al., 2012; Siles and Margesin, 2016) while (Jassey et al., 2013) found the highest bacterial diversity at medium elevations and Singh et al. (2014) found a higher bacterial diversity at higher altitudes than at medium altitudes.

The aforementioned studies have provided some valuable knowledge about forest soil microbial community, but some key information about the effect of native forest *vs*. plantation forest and forest age on soil microbial community are lacking. Furthermore, while native forests represent model systems for studying processes which are important for the implementation of sustainable forest management (Hackl et al., 2004; Zimmerman and Kormos, 2012), it can also be used to provide baseline information about soil microbial diversity and composition in unmanaged forest ecosystems. However, to the best of our knowledge, there is no single study that investigated a comparative comprehensive microbial analysis of forest soils from plantation forest *vs*. a native forest ecosystem in the tropics. This is crucial to assist with forecasting forest ecosystem attributes, processes and impact of forest type on soil physical-chemical and microbial community in the tropics.

Trinidad and Tobago is heavily forested and most of the *ca*. 230,000 ha of forest is state-owned. Commercial Teak plantations were established in 1925–1927 with the introduction of monoculture stands of teak (*Tectona grandis*), and Teak timber is now an important commercial forest product in Trinidad. However, while forestry is important to Trinidad and Tobago, there has not yet been any investigation of microbial communities in these ecosystems and their potential linkages to tree species and forest ecosystem function. Hence, to gain a better understanding of how microbial communities respond to changes in forest vegetation, the objectives of this study were: (1) To determine if there are any significant differences between the microbial alpha- and beta- diversities of a plantation forest (teak) soil vs. a native forest soil, (2) To determine if there are significant effects of forest type, age of teak stands and forest location on microbial biodiversity of soils, (3) To determine if there are any changes in the levels and patterns of key soil enzymes in a plantation forest (teak) soil *vs*. a native forest soil.

The hypotheses tested in this study were: (1) Microbial (bacterial and fungal) alpha- and beta diversities of teak plantation soils would be significantly reduced relative to that of the original diverse native forest soil, (2) There are no significant effects of forest type, age of teak stands and forest location on microbial biodiversity of soils, and (3) Levels and patterns in the activity of key soil enzymes would be altered in soils shift to Teak plantation relative to that of the original diverse native forest. Hence, a polyphasic approach employing microbial community characterization via high-throughput Illumina sequencing of prokaryotic and eukaryotic communities as well as by PLFA analysis, and enzymatic activity was conducted.

## Materials and methods

### Site description, sampling plan, and soil sampling

The study was done at two locations in Trinidad (10° 3′N 60° 55′W and 10° 50′N 61° 55′W) on forests owned by the Ministry of Agriculture (Figure S1). Investigations were conducted on Teak plantations (T) and adjacent native forests (control) in the Tamana site (hereafter referred to as the north location) and the Brickfield, Tabaquite site (hereafter referred to as south location). Briefly, Teak is a deciduous tree and belongs to the *Lamiaceae* family. The native north and south stands were both dominated by *Spondias monbin* (Hog Plum), *Brownea latifolia* (Cooper Hoop), *Pentaclethra macroloba* (Bois multare), *Carapa guianensis* (Crappo) and *Trichilia oblanceolata* (Acurel). Additional tree species in the North were *Tabebuia serratifolia* (Yellow pouis), *Swartzia pinnata* (Bois pois), *Eschweilera subglandulosa* (Guatecare), *Manilkara* balata (Balata) and *Sterculia caribaea* (Mahoe). In the south, additional species were: *Pachira insignis* (Wild chataigne), *Trichilia trinitensis* (Obi), *Pisonia eggersiana* (Jiggerwood) and *Isertia parviflora* (Bois Fer).

Teak stands of three age groups were analyzed. One group was the oldest Teak trees that were established in 1943 and located only at the south site. A second set was middle-aged plantations that were established between 1956 and 1968 at both the north and south locations. The third set was a stand of young trees that was established in 2009 at the north site only. Hereafter, the Teak stands are abbreviated as from the North (N) or South (S) locations preceded by the year of establishment: 1957 (57N), 1966 (66N), 2009, (09N), 1943 (43S), 1956 (56S), 1968 (68S). Native forests from the north and south locations are designated as NatN and NatS, respectively.

Within each selected stand, soil samples and associated data were taken on a randomly determined line segment. For each stand, the length of the line transect was 200 m and five sampling points were determined systematically. Five replicate composited samples from each stand were obtained at a distance 40 m apart. At each of the five sampling point, a soil auger was used to collect five soil samples from the top 0 to 20-cm (A horizon) thus giving 25 soil samples. Hence, each of the five sample points consisted of a composite of five soil samples which were kept separate for all further analyses. Samples were transported to the laboratory where they were homogenized by 2-mm sieving, and fine roots were removed. Samples were then partitioned for analysis of enzymatic activity, PLFA extraction, DNA extraction, and edaphic characterization. Enzyme analysis and pH measure was done on all five of the samples. The remainder of the analyses was done on three samples randomly chosen from the set of five.

### Soil physicochemical analyses

Soil pH was determined by the slurry method (1:5, w:v; sample:distilled deionized water) measured with an Eijkelkamp pH/mV/EC/Salinity/T/02m (Agrisearch Equipment ZG Giesbeek, the Netherlands). Total carbon (TC), organic carbon (OC), and inorganic carbon were determined by dry combustion using a LECO CNS-2000 analyzer. Total N (TN) was determined by combustion and by the Kjeldahl digestion method (TKN). Nitrate-N was measured in soil water extracts colorimetrically after reaction with phenoldisulphonic acid, and ammonium-N was determined by flow injection analysis. Major and minor trace elements were determined by inductively-coupled mass spectrometry. Cation exchange capacity (CEC) was determined by ammonium acetate extraction proceeded by cation quantification via atomic absorption spectrometry. Soil physicochemical characteristics are displayed in Table 1 and Table S1.

**Table 1:** **Characteristics of selected soil properties**.

**Forest stand**	**NH_4_-N (mg/kg)**	**NO_3_-N (mg/kg)**	**TN**	**TKN**	**Sand**	**Silt (%)**	**Clay**	**Total Carbon**	**CEC (cmol (+)/kg)**	**TC:TN**	**TC:TKN**	**pH**
1957N	14.35 ± 0.7	22.43 ± 0.8	0.26 ± 0.8	0.25 ± 0.6	31.33 ± 0.7	44.67 ± 0.7	30.67 ± 0.7	2.28 ± 0.7	10.22 ± 0.8	8.77 ± 0.7	9.21 ± 0.6	4.80 ± 0.5
1966N	39.17 ± 0.7	45.10 ± 0.7	0.39 ± 0.8	0.43 ± 0.7	18.00 ± 0.9	25.00 ± 0.8	57.00 ± 0.6	3.72 ± 0.4	5.93 ± 0.8	9.62 ± 0.7	8.73 ± 0.5	4.10 ± 0.5
2009N	23.62 ± 0.8	20.38 ± 0.7	0.27 ± 0.6	0.27 ± 0.8	7.33 ± 0.8	45.33 ± 0.8	47.33 ± 0.8	1.98 ± 0.7	5.15 ± 0.8	7.44 ± 0.8	7.29 ± 0.7	4.40 ± 0.7
NatN	31.00 ± 0.6	21.35 ± 0.8	0.26 ± 0.8	0.26 ± 0.7	11.67 ± 0.8	39.67 ± 0.6	48.67 ± 0.8	1.98 ± 0.7	4.05 ± 0.6	7.66 ± 0.7	7.67 ± 0.7	4.10 ± 0.6
1943S	43.72 ± 0.8	0.98 ± 0.6	0.31 ± 0.8	0.31 ± 0.9	8.67 ± 0.7	40.33 ± 0.0.8	51.33 ± 0.8	2.89 ± 0.6	9.14 ± 0.8	9.34 ± 0.4	9.21 ± 0.8	4.20 ± 0.7
1956S	25.17 ± 0.7	9.77 ± 0.9	0.27 ± 0.7	0.30 ± 0.8	28.00 ± 0.9	33.67 ± 1.6	39.00 ± 0.8	2.43 ± 0.6	6.18 ± 0.5	9.04 ± 0.7	8.06 ± 0.7	4.80 ± 0.8
1968S	24.63 ± 0.7	26.12 ± 1.1	0.35 ± 0.8	0.37 ± 0.8	23.00 ± 1.1	27.00 ± 0.7	50.00 ± 0.7	3.38 ± 0.6	11.21 ± 0.7	9.71 ± 0.8	9.25 ± 0.5	5.00 ± 0.7
NatS	8.19 ± 0.7	81.00 ± 1.2	0.56 ± 0.7	0.44 ± 0.7	14.33 ± 0.9	15.33 ± 0.6	69.67 ± 1.1	5.64 ± 0.4	23.03 ± 0.7	10.04 ± 0.9	12.82 ± 0.8	5.20 ± 0.8

*TN, Total Nitrogen; TKN, Total Kheldhal Nitrogen; NH_4_-N, Ammonium Nitrogen; NO_3_-N, Nitrate Nitrogen; CEC, Cation Exchange Capacity, values are average ± standard deviation*.

### Extracellular enzyme activity (EEA)

EAA was conducted according to Saiya-Corka et al. (2002). Soil (1 g wet weight) was added to 125 mL sodium acetate buffer (50 mM, pH 5) and homogenized in a Waring 7009G blender (Waring Laboratory Science, Torrington CT) run at top speed (22,000 RPM) for 1 min. The soil slurry was then decanted into a flat bottom tray containing a stir bar for rapid mixing to maintain the soil suspension, and 12 aliquots (200 μl each) were transferred simultaneously to a 96-well microplate (Thermo Fischer Scientific, Waltham, MA) by using a multichannel pipet. Activities of seven EEA were assayed (Table 2), which represented key reactions in carbon, nitrogen and phosphorus cycles; all EEA were measured by using substrates that generated a fluorescent product, either 4-methylumbellyferylone (MUB) or 7-amino-4-methylcoumarin (AMC). All substrates were obtained from Sigma Aldrich Chemicals (St. Louis, MO). Stocks (20 mM) of all substrates were made in dimethyl sulfoxide (DMSO, Sigma Aldrich) except 4-MUB-β-D-xylopyranoside, which was dissolved in pyridine (Sigma Aldrich). The working solutions were created by diluting all stocks to 200 μM (in DMSO or pyridine as appropriate), 50 μl of which was added to each assay giving a final substrate concentration of 40 μM. Fluorescence was measured at time zero, and after 3 h incubation in the dark at 30°C by using a Synergy plate reader (BioTek Instruments, Winooski, VT) with excitation = 365 nm, emission = 450 nm, and the chamber held at 30°C. Product formation was quantified by reference to six point standard curves (100 μM to 1 μM) of MUB or AMC in the assay buffer. The MUB and AMC standard curves were also established in 200 μl aliquots of the soil suspensions as quench controls. Blanks included: assay buffer alone, substrates in buffer alone, and soil suspension in assay buffer alone. For each soil sample, the 12 replicate fluorescence values obtained for each substrate were averaged, corrected for quenching and background, and rates of product formation calculated that were normalized to oven dry weight of soil (nmol/h/g soil).

**Table 2:** **Enzymes assayed and substrates**.

**Enzyme**	**EC^a^**	**Substrate^b^**
N-Acetlyglucosaminidase	3.2.1.52	4-MUB-N-acetylglucosaminide
Cellobiohydrolase	3.2.1.91	4-MUB-N-cellobiopyranoside
α-Glucosidase	3.2.1.20	4-MUB-α-D-glucopyranoside
β-Glucosidase	3.2.1.21	4-MUB-β-D-glucopyranoside
Leucine aminopeptidase	3.4.11.1	L-leucine-7-amido-4-methylcoumarin
Phosphomonoesterase (Acid)	3.1.3.2	4-MUB-phosphate
β-Xylosidase	3.2.1.37	4-MUB-β-D-xylopyranoside

a *EC, Enzyme Commission number*.

b *MUB, methylumbellyferyl*.

### Phospholipid fatty acid analyses

Phospholipid fatty acids (PLFA) were extracted from soil by using a Bligh and Dyer technique (Bligh and Dyer, 1959) as modified as described by (Moeskops et al., 2010). Briefly, each soil sample (2 g) was extracted with a chloroform–methanol–citrate buffer mixture (1:2:0.8 v/v/v), and then fractionated into neutral, glycol, and phospholipids by passage over a silicic acid column (SPE-SI; Bond Elut 3CC, Varian, Inc.). Phospholipids were then subjected to mild alkaline methanolysis for esterification of fatty acids. The extracts were analyzed by gas chromatography-mass spectrometry by using a Hewlett–Packard 6890 fitted with a DB-5 ms column (Agilent, Santa Clara, CA) interfaced to a Hewlett–Packard 5973 mass selective detector. Peak identification was done referencing retention times and mass spectra; quantification was done by using 19:0 as an internal standard. A total of 86 PLFA were identified, and up to 51 of these with carbon chains ≤C20 were used for biomass quantification. Selected PLFA were used to quantify specific guilds. Gram-positive (GP) bacteria were quantified by the sum of 15:0 ISO, 15:0 ANTEISO, 17:0 ISO, and 17:0 ANTEISO. Gram negative (GN) bacteria were quantified by the sum of PLFA 16:1ω7*c*, 17:0 CYCLO, 18:1 ω5*c*, 18:1 ω7*c*, 19:0 CYCLO and 19:0 CYCLO 11-12 2OH. The sum of 16:0 10 Methyl and 18:0 10 Methyl was used to quantify *Actinomycetes*. Fungal quantification was done with the sum of 16:1 ω5*c*, 18:1 ω9*c*, and 18:2 ω6,9*c* with the former used to quantify the subset of arbuscular mycorrhizal fungi (AMF). The fungi:bacteria ratio calculation was based on the sum of all fungal PLFA listed above, divided by the sum of the above-listed PLFA used for quantification of GN- and GP-bacteria (Joergensen and Wichern, 2008).

### DNA extraction, sequencing, and sequence database processing

Three DNA extractions were prepared from each soil in batches of 0.25 g, and a 1 uL aliquot from each of the 0.75 g-equivalent extracts was used for generation of bacterial and fungal amplicon libraries. Bacterial libraries were created with universal prokaryote primers 515F and 806R that targeted the V4–V5 region of the 16S rRNA gene (Caporaso et al., 2010), amplicons were generated following PCR protocols described in the work referenced as the source of primers. Fungal libraries were created with primers ITS1F (Gardes and Bruns, 1993) and ITS4 (White et al., 1990) following the PCR protocol described by Manter and Vivanco Manter and Vivanco (2007). Region specific primers were modified to add Illumina adapter overhang nucleotide sequences to the amplicons. Following initial amplification, library size was verified on an Agilent DNA1000 chip, cleaned using a 1X volume of Mag PCR clean-up beads (Axygen Biosciences, Union City, CA) and then Illumina dual indexes and sequencing adapters were added by PCR (de Gannes et al., 2015). Following PCR, samples were cleaned and normalized by using a Sequal Prep Normalization Plate (Life Technologies, Carlsbad, CA). Quality and quantity of the libraries were assessed by using an Agilent DNA1000 chip and Qubit® dsDNA HS Assay Kit, respectively, and were standardized to 2 nM prior to pooling and sequencing. Sequencing was done with an Illumina MiSeq system (Illumina, San Diego, CA) by using Miseq reagent kit v. 3 (Illumina) to generate 2 × 250 bp paired end reads. Images were analyzed using the standard Illumina Pipeline, version 1.8.2.

Illumina datasets were de-multiplexed by using MiSeq Reporter v2.2.31. (Illumina) with a Q20 minimum value as a quality filter, and then reads trimmed of forward and reverse primers by using cutadapt (Martin, 2011). For bacterial libraries, paired sequences were merged into single reads by using FLASH (Magoč and Salzberg, 2011), and then length-filtered. The fungal ITS extractor was applied to fungal libraries to isolate ITS sequences by removing adjoining regions encoding ribosomal RNA (Nilsson et al., 2010). The QIIME (Quantitative Insights into Microbial Ecology) package (v. 1.8.0) was then utilized for generation of operational taxonomic units (OTU) generation by picking with against Greengenes (v. 2013_08), and supplemented by *de novo* clustering at 97% similarity. The sequence libraries generated by the forward and reverse ITS primers were maintained as separate data sets. The fungal community analyses was done using sequences from the forward primer, which covered ITS1 since there has been a larger use of that region in the literature (Chen et al., 2001; Narutaki et al., 2002; Hinrikson et al., 2005; Nilsson et al., 2008).

For bacterial libraries, taxonomy was assigned by BLAST against GreenGenes (v. 2013_08) and the libraries were screened for chimeric reads by using Chimeraslayer against GreenGenes (v. 2013_08). For fungal libraries, the UNITE database (alpha version 2012_11) was used for taxonomic assignment of OTU by BLAST, and for chimera screening. The QIIME package (v. 1.8.0) was used for OTU-based analyses including rarefaction and computation of diversity metrics (excluding singletons). For comparisons between samples, libraries were rarefied to a common number of sequences for the different taxonomic groups, which corresponded to the smallest library (Figure S1). Prism 6 (Graphpad, La Jolla, CA) was used to display the composition of each library by using average relative abundance for taxa believed to be best able to convey biologically relevant information.

### Statistical analyses

The R programming environment (www.r-project.org) was used for univariate analyses including analysis of variance (ANOVA), and pairwise comparison (Tukey's test). False discovery rates were determined by the method of Benjamini and Hochberg (1995) and significance was assessed by setting the resulting *q*-values ≥ 0.05. Multivariate analyses were done by using Primer-E v. 6 (PRIMER-E Ltd, Lutton, UK). For Illumina libraries, the numbers of reads assigned to an OTU were normalized to the total reads in a given library. Each dataset was square root transformed to down weight effects of highly abundant OTU and then a resemblance matrix constructed based on Bray-Curtis similarities (Clarke et al., 2008). Bray-Curtis similarity matrices were also constructed on PLFA data that was square root transformed to down weight importance of highly abundant individual PLFA. The Illumina and PLFA matrices were analyzed by non-metric multidimensional scaling (NMDS) ordination (25 restarts, minimum Kruskal stress = 0.01). Similarity contours overlaid on NMDS ordinations were based on groupings developed with the CLUSTER routine. The matrices were analyzed by two-way analysis of similarity (ANOSIM, tests the null hypothesis: the average of the ranks of within-group distances is greater than or equal to the average of the ranks of between-group distances) using both nested and crossed designs. Nested ANOSIM was first applied to determine if there were significant differences between Teak stands using the factors “stand” (the different teak stands according to age, selected for the study) and “location” (North or South). Crossed analysis examined the data for differences between the Teak plantations as a group compared to the native forest as a group, using the factors “forest type” (either the plantation teak forest or the natural forest) and “location.” Any factors determined to be significant by ANOSIM were then examined by canonical analysis of principal components (CAP) and ordination; CAP trace statistics were determined by 999 permutations. Relationships between beta-diversity patterns and environmental data were examined by using two non-parametric tests, the RELATE and the Bio-environmental Step (BEST) routines. RELATE is a Mantel-type test that generates a Spearman Rho test statistic that measures congruence between matrices of biotic or abiotic data. BEST identified subsets of physicochemical variables that gave rank order similarities (Euclidean distance) between soils that best matched the rank order Bray-Curtis similarities of microbial community composition (Clarke et al., 2008).

### Sequence accession numbers

The Illumina sequence data reported here is deposited in the NCBI Sequence Read Archive (http://www.ncbi.nlm.nih.gov/sra) under accession number SRP058555.

## Results

### EAA

**Figure 1:**
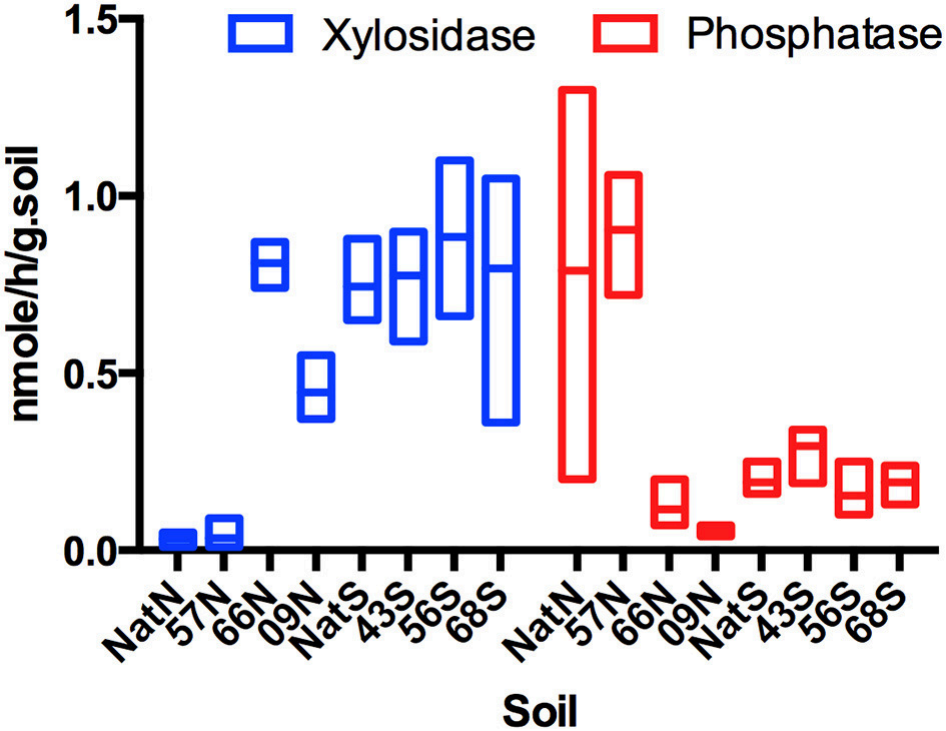
**Box Plot of xylosidase and phosphatase activities (nmole/h/gram.Soil) across forest soils**. Letters indicate forest soils sample sites followed by year of establishment and are abbreviated as: N, North; S, South and Nat, Native.

Levels of β-xylosidase were similar in the north native forest and 57N samples, but were significantly (*F* = 5.29, *p* = 0.0163) lower than that of all other samples (Figure 1). At the north location, β-xylosidase activity was highest in the 66N sample, which was significantly greater (*F* = 4.68, *p* = 0.0113) than that of 09N (Figure 1). At the south location, β-xylosidase activity did not vary significantly among samples, and levels were on average similar to that of 66N. The north native forest and 57N samples were also similar in having comparatively high levels of phosphomonoesterase (acid phosphatase), which were significantly greater (*F* = 5.33, *p* = 0.0313) than those of 09N or 66N samples (Figure 1). Again, in the south samples, there were no significant differences in acid phosphatase activity, and levels were on averaged similar to those in the 09N and 66N samples. For the other four EAA assayed, there were no significant differences in activity in any of the samples.

### PLFA analyses: biomass and community structure

Biomass levels based on PLFA content differed significantly (ANOVA *F* = 5.59, *p* = 0.0192) between the north stands (Table 3). The specific stand pairs from the north that showed significant differences (Tukey test *p* < 0.05) were: 57N vs. 09N, native vs. 57N and native vs. 09N. While these differences were detected, there were no apparent trends: soil biomass was not consistently higher or lower in Teak *vs*. native forest, and among Teak stands, biomass was not consistently higher or lower as a function of stand age. Microbial biomass levels in the south location were on average significantly greater than those of the north (Table 3; ANOVA *F* = 7.43, *p* = 0.021) but there were no significant differences between south stands in the amounts of microbial biomass (ANOVA *F* = 1.12, *p* = 0.511).

**Table 3:** **Soil microbial biomass and guilds based on PLFA analysis**.

**Stand**	**Biomass**	**Fungi**	**AMF**	**GN**	**GP**	**Actinomycete**
**NORTH**						
2009N	0.366 ± 0.064	14.00 ± 1.89	3.95 ± 0.62	11.99 ± 0.86	14.13 ± 0.79	5.80 ± 0.54
1957N	0.343 ± 0.040	16.17 ± 1.57	4.23 ± 1.18	10.18 ± 1.84	10.18 ± 2.46	4.47 ± 0.75
1966N	0.557 ± 0.047	10.65 ± 1.2	3.07 ± 0.25	11.9 ± 0.79	13.22 ± 0.87	4.66 ± 0.29
NatN	0.363 ± 0.051	12.00 ± 0.55	3.68 ± 0.17	10.92 ± 0.91	14.01 ± 0.87	5.71 ± 0.36
All North	0.403 ± 0.105	13.21 ± 1.53	3.73 ± 0.64	11.25 ± 0.43	12.89 ± 0.70	5.16 ± 0.18
**SOUTH**						
1943S	0.621 ± 0.024	19.64 ± 2.63	4.82 ± 0.16	9.44 ± 0.65	10.33 ± 0.46	4.01 ± 0.22
1956S	0.445 ± 0.169	11.59 ± 1.78	3.91 ± 0.23	11.59 ± 1.18	12.51 ± 1.45	4.71 ± 0.58
1968S	0.601 ± 0.075	11.5 ± 2.94	4.97 ± 0.54	11.5 ± 0.81	12.00 ± 1.22	4.99 ± 0.54
NatS	0.660 ± 0.125	12.14 ± 0.62	3.84 ± 0.58	12.14 ± 0.62	14.98 ± 0.50	6.45 ± 0.62
All South	0.582 ± 0.139	13.72 ± 0.90	4.39 ± 0.18	11.17 ± 0.58	12.46 ± 0.73	5.04 ± 0.16

*Values are average μmol PLFA/g soil 1 standard deviation. See text for specific PLFA used in the calculation of each category*.

**Figure 2:**
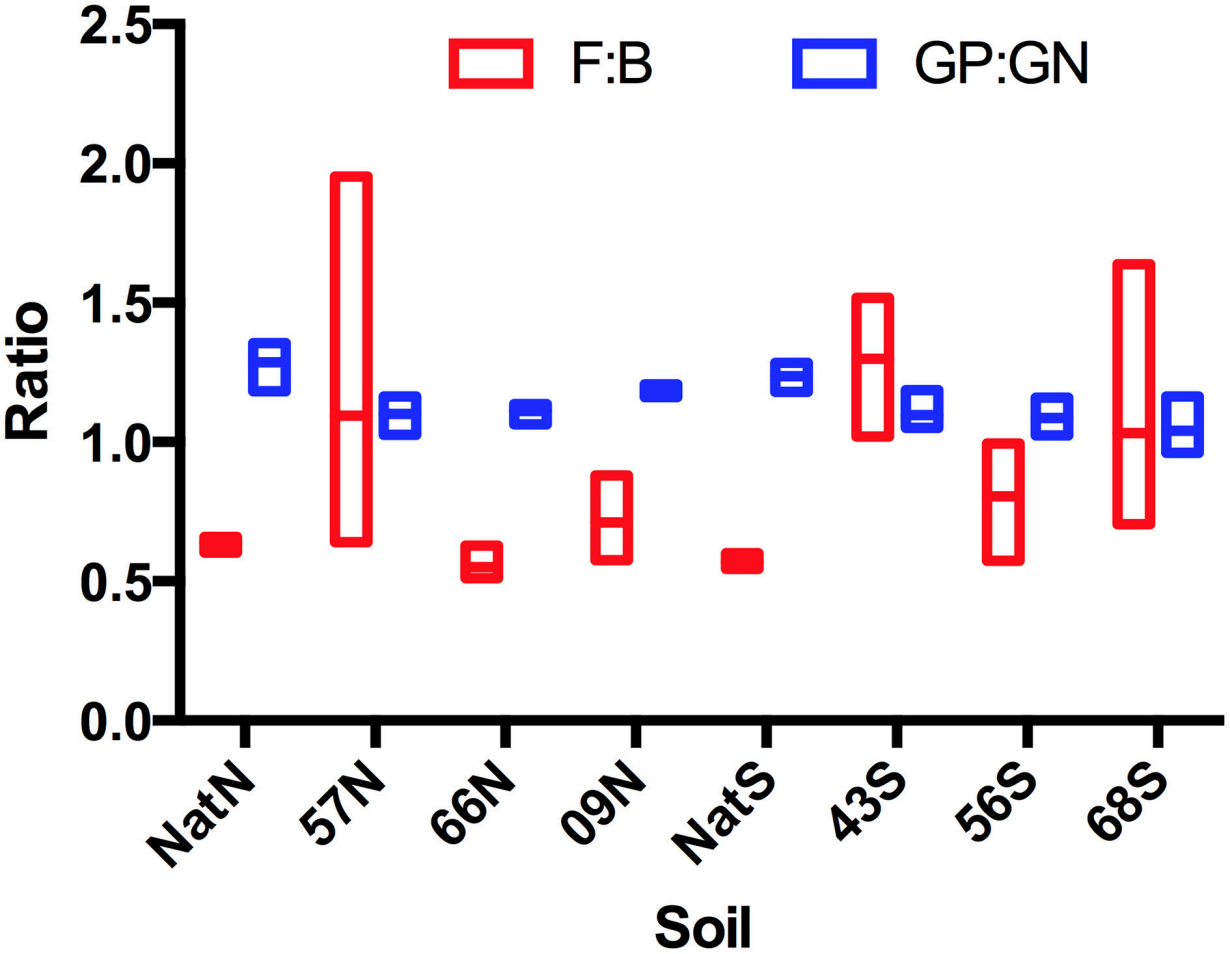
**PLFA-based community structure illustrating ratios of microbial guilds**. Abbreviations represent: year of establishment; Nat, Native; North, N and S, South; F, fungi, B, bacteria; GP, gram positive bacteria; GN, gram negative bacteria (GP:GN).

For PLFA-based community structure analysis, two broad indicators used were the ratio of fungi to bacteria (F:B) and the ratio of GP bacteria to GN bacteria (GP:GN). Neither of these ratios differed significantly among soils from native forest or Teak stands at either location. The main trend apparent from the data was the much greater dispersion of F:B values in the Teak stands relative to the native forest (Figure 2). For native stands, all F:B were in a relatively narrow range and all were <1. Teak stands had a much wider range with some >1. At the north, there were no significant differences among stands in the abundance of individual guilds assayed by PLFA (Table 3). However, at the south location, there were significant differences between soils in the abundance of *Actinomycetes* (*F* = 8.01, *p* = 0.0086), fungi (ANOVA *F* = 6.00, *p* = 0.0191), and GP bacteria (ANOVA *F* = 3.95, *p* = 0.0532). But, as with biomass, while differences were detected, there were no apparent trends as a function of forest type or of the age of Teak stands. The exception was the *Actinomycetes*, which, at the south location, were significantly less abundant in all Teak soils than in the soils of the native forest (ANOVA *F* = 6.97, *p* = 0.002).

**Figure 3:**
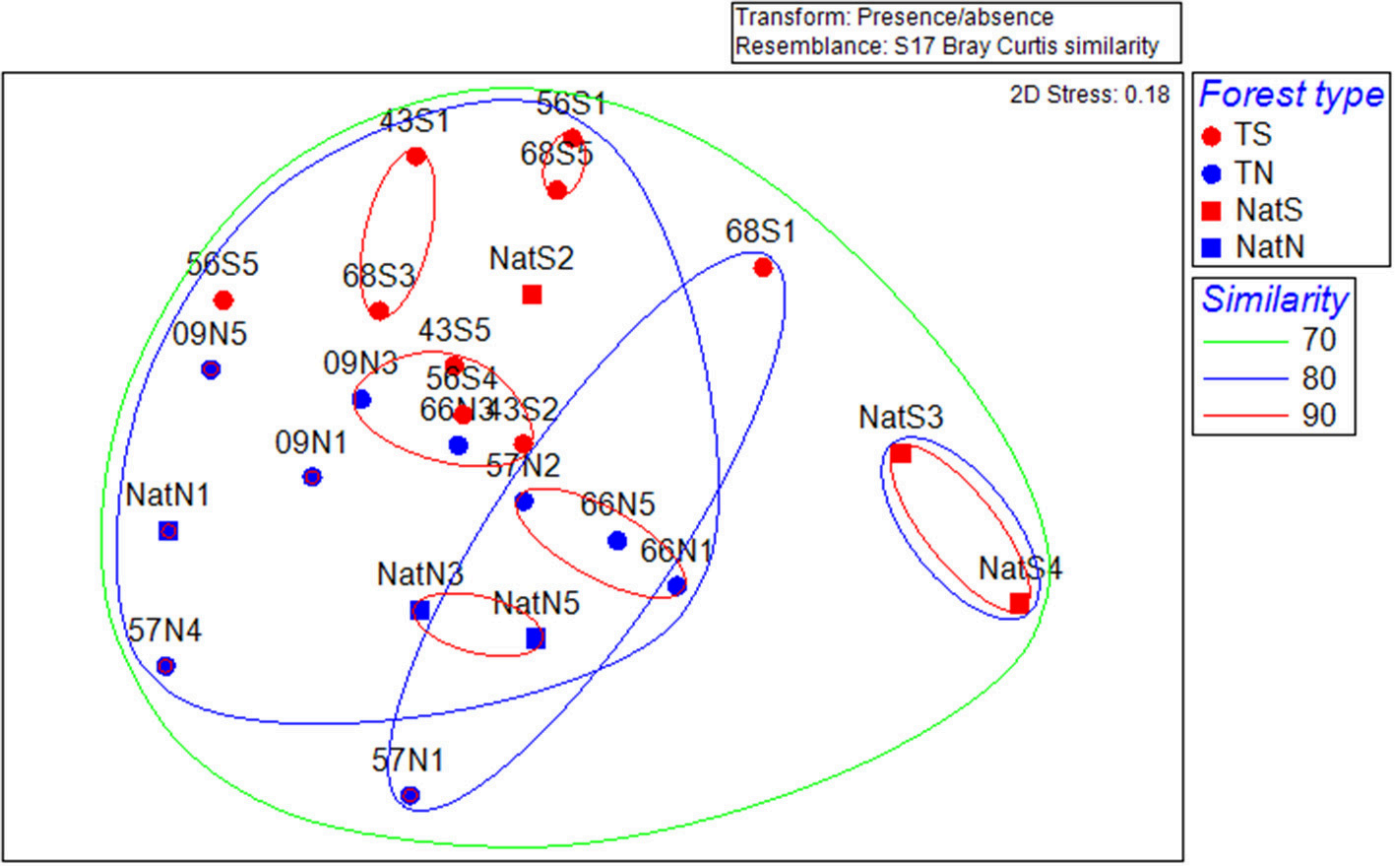
**Non-metric multidimensional scaling ordinations for separation of microbial community structure by location based on PLFA data. T, Teak; S, South; N, North; Nat, Native**. Numbers indicate replicate number. Dotted lines are percent similarity contours derived from cluster analysis.

Multivariate analysis of community structure based on PLFA data included unconstrained ordination by NMDS. The primary trend illustrated by this analysis was separation of microbial communities by location; there was no apparent clustering by stand type or by stand age in the case of the Teak samples (Figure 3). CLUSTER analysis revealed that the structure of microbial communities was 80% similar across all soils. Two-way nested ANOSIM examining differences among Teak stands showed that only location was significant (stand, *p* = 0.069; location, *p* = 0.01) while two-way crossed ANOSIM showed that both forest type and location were significant (forest type, *p* = 0.044; location, *p* = 0.01). CAP analysis using forest type as a factor gave strong canonical correlations (δ_1_ = 0.89, δ_2_ = 0.87) and by permutation yielded a highly significant first squared canonical correlation ($\delta_{1}^{2}$ = 0.789, *p* = 0.004) and a highly significant trace statistic (1.157, *p* = 0.001). In this constrained ordination, the first canonical axis (CAP1) separated soils by forest type, with native forest soils segregated to the left and Teak soils segregating to the right (Figure S2). The second canonical axis (CAP2) separated samples by location with the north and south samples segregating to the bottom and top of the axis, respectively (Figure S2).

Potential correlations of variation in PLFA-based community structure to edaphic properties were also examined by using the RELATE and BEST tests. The former analysis gave a Rho value of moderate strength (0.413) but which was strongly significant (*p* = 0.001). The BEST analysis gave a single correlation identified the parameters most closely associated (0.56) but which was strongly significant (*p* = 0.009) included six variables: TC, TN, NH_4_-N, NO_3_-N, CEC, and Mn.

### Illumina sequence analysis of 16S rRNA (bacteria) and ITS (fungi) amplicon libraries: microbial diversity and community structure

Rarefaction curves showed that the sequencing work was relatively comprehensive to cover bacterial and fungal diversity as the rarefaction curves tended to approach saturation plateau (Figures S3, S4).

**Figure 4:**
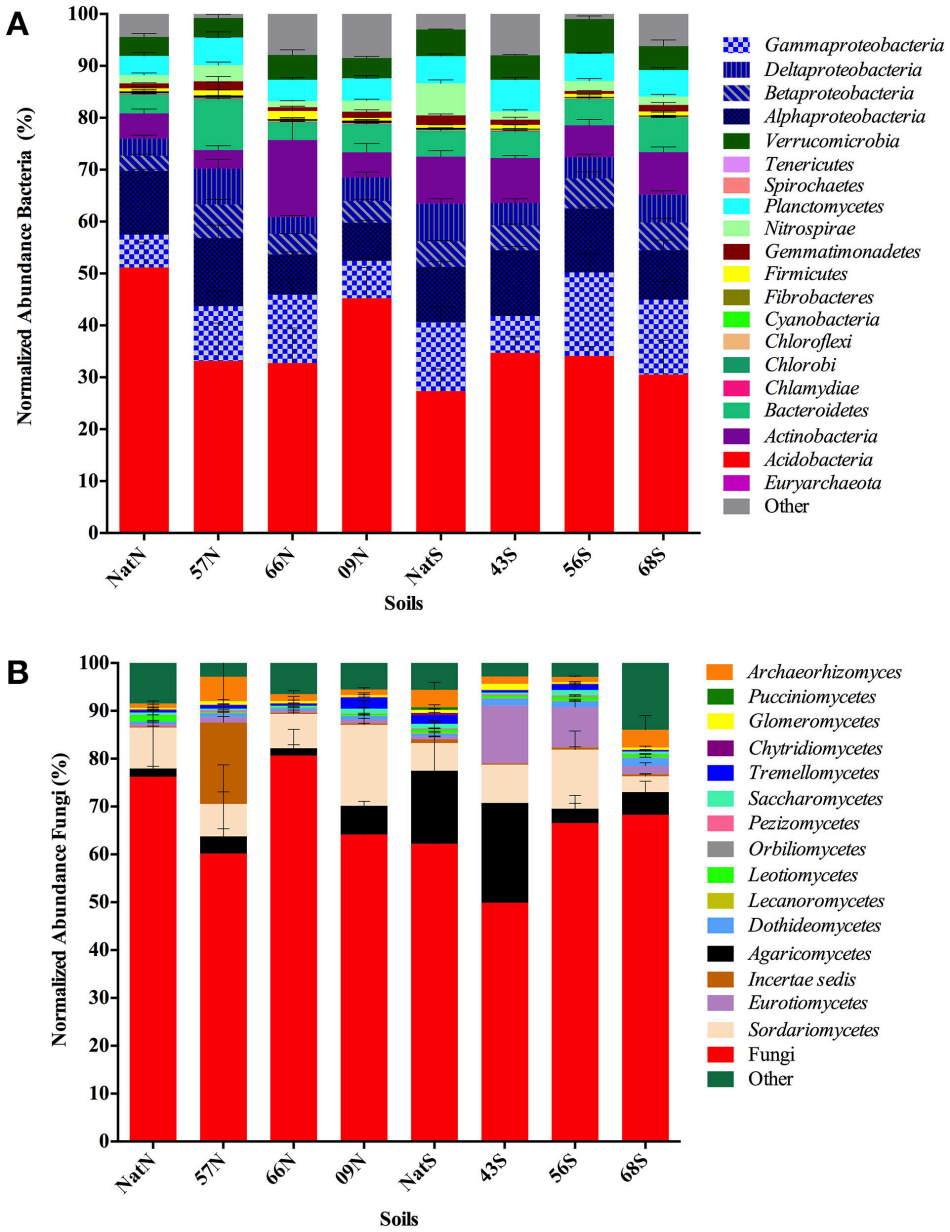
**(A)** Comprehensive view of the sequence content of forest soil libraries for prokaryotes. Segments composing each bar are mean number of sequences in the indicated taxa normalized to the total number of sequences in each library. Standard error of each mean is indicated by lines within each segment. Letters indicate forest soils sample sites followed by year of establishment and are abbreviated as: T, Teak; S, South; N, North; Nat, Native. **(B)** Comprehensive view of the sequence content of forest soil libraries for Fungi. Segments composing each bar are mean number of sequences in the indicated taxa normalized to the total number of sequences in each library. Standard error of each mean is indicated by lines within each segment. Letters indicate forest soils sample sites followed by year of establishment and are abbreviated as: T, Teak; S, South; N, North; Nat, Native.

A total of 879 OTU were identified with six OTU assigned as *Archaea* and 872 OTU assigned as *Bacteria* (Table S2). For the bacterial community, 20 taxa accounted for the majority of sequences (Figure 4A). The *Acidobacteria* predominated across all soil samples (28–51%), followed by *Proteobacteria* (*Gammaproteobacteria*, 6–16%; *Alphaproteobacteria*, 9–13%; *Deltaproteobacteria* and *Betaproteobacteria*, (3–7%). Other top taxa were: *Actinobacteria* (4–15%), *Bacteriodetes* (4–10%), *Verrucomicrobia* (4–7%), and *Plantomycetes* (4–6%). Four phyla differed significantly between the native soils and those in the Teak plantations: those that were significantly more abundant in the native soils than in soils of Teak plantations were *Cyanobacteria* (ANOVA *F* = 6.54, *p* = 0.0180) and *Nitrospirae* (ANOVA *F* = 7.96, *p* = 0.0100) while those that were significantly more abundant in Teak soils than in soils of the native forest were candidate phylum TM7 (ANOVA *F* = 17.14, *p* = 0.0004) and candidate phylum WS6 (ANOVA *F* = 4.54, *p* = 0.0445).

For bacteria, the top two most abundant OTU were the same in all soils, being identified as *Acidobacteria* either to the order Ellin6513 (most abundant OTU) or to the family level as *Koribacteraceae* (second most abundant OTU; Table S2). Besides the top two OTU, the soils were further similar in that same collection of OTU comprised were most abundant in all samples although the specific order of abundance varied by soil. This group included OTU identified as (listed in order of decreasing average abundance across all soils): *Acidobacteria* order iii1-15, *Bacteroidetes* family *Chitinophagaceae, Alphaproteobacteria* family *Rhodospirillaceae*, Planctomycetes, order WD2101, *Acidobacteria* genus Candidatus Koribacter, *Acidobacteria* genus Candidatus Solibacter, *Acidobacteria* class *Acidobacteria*-5, *Alphaproteobacteria* genus *Rhodoplanes* and *Verrucomicrobia* genus DA101.

For the fungi, a large group of sequences (49–80%) were assigned only to the *Fungi* domain level (Table S3; Figure 4B). At the level of class, the *Sordariomycetes* predominated (3–16%) followed by the *Agaricomycetes* (2–21%). The *Eurotiomycetes* were prevalent in two of the south soils 43S, (12%) and 56S (8%). There were no significant differences in abundance of any of the fungal classes, except for one unidentified *Glomeromycota* class, which was significantly more abundant in Teak soils than in soils of the native forest (ANOVA *F* = 4.47, *p* = 0.047). A total of 793 OTU were identified, with **one** OTU (assigned only as *Fungi*) dominant across all soils comprising 29–79% of the total reads in a given library. As with the bacteria, the fungal libraries of all soils were composed of a few common OTU, which were identified as (listed in order of decreasing average abundance across all soils): *Basidiomycota* genus *Hygrocybe, Ascomycota* class *Sordariomycetes, Basidiomycota* genus *Sparassis* and as the phylum *Ascomycota*.

**Figure 5:**
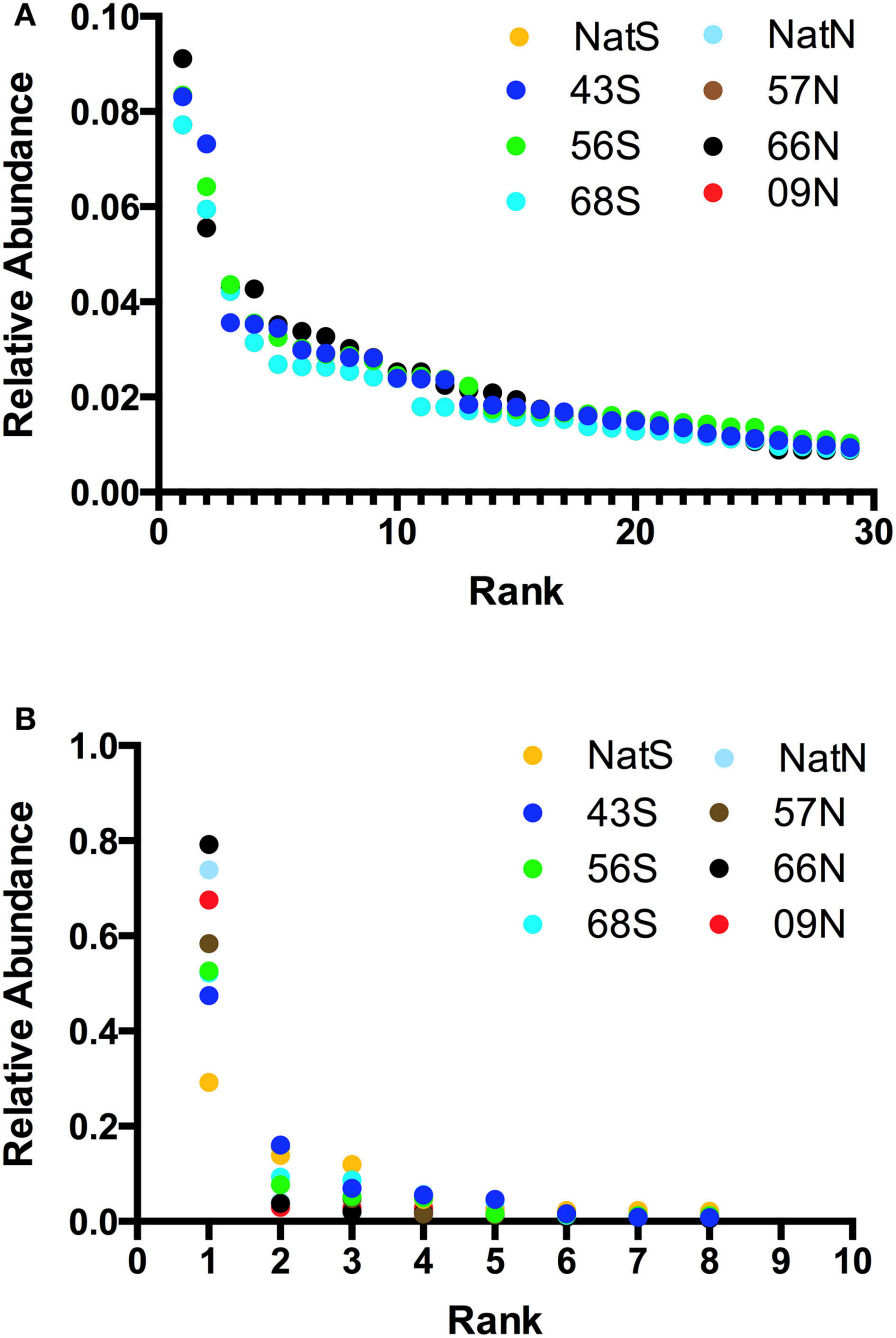
**(A)** Alpha-diversity characteristics visualized on Whittaker plot for bacterial community, where line slopes reflect species (OTU) evenness and line lengths indicate OTU richness. Letters indicate forest soils sample sites followed by year of establishment and are abbreviated as: T, Teak; S, South; N, North; Nat, Native. The values plotted are the average fractional abundance in each library. All values are plotted but some overlap and hence are invisible. **(B)** Whittaker plots illustrating domination of all fungal communities by a single OTU. Letters indicate forest soils sample sites followed by year of establishment and are abbreviated as: T, Teak; S, South; N, North; Nat, Native. The values plotted are the average fractional abundance in each library. In both panels relative abundance is the fraction of all sequences in a given library that were represented by a single OTU.

Alpha-diversity parameters (OTU richness and evenness) did not differ significantly (*p* = 0.748) between the bacterial communities as reflected in the Chao1 metric. Alpha-diversity characteristics were visualized with Whittaker plots, where line slopes reflected species (OTU) evenness and line lengths indicated OTU richness, which were similar for all soils (Figures 5A,B). The fungal communities also showed no significant differences (*p* = 0.627) in Chao1. Likewise, Whittaker plots displayed similar patterns of fungal diversity in all of the soils (Figure 5B). The domination of all fungal communities by a single OTU was readily apparent from the Whittaker plots (Figure 5B); this OTU was the same in all soils and was identified only to the kingdom level as *Fungi*.

**Figure 6:**
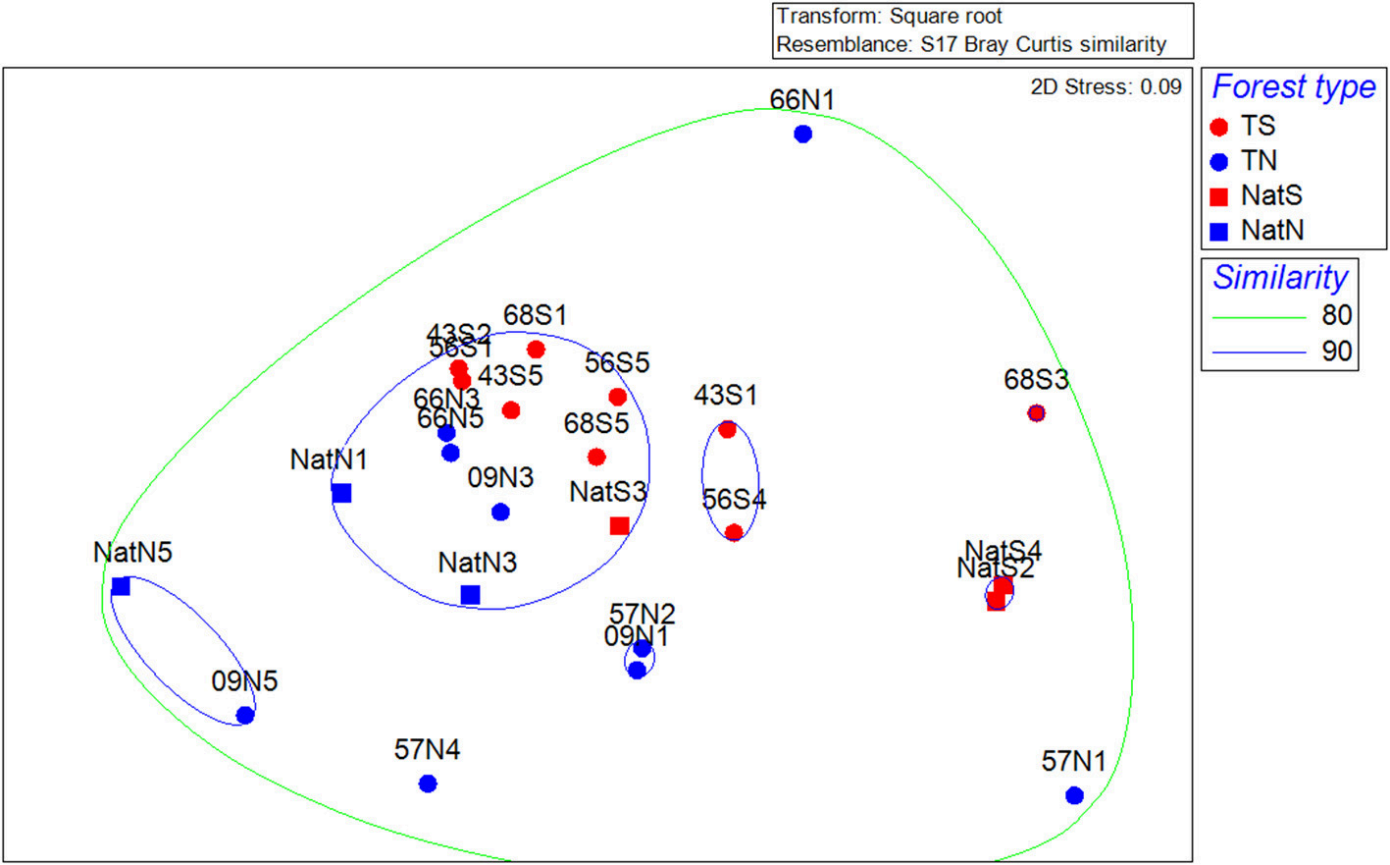
**Non-metric multidimensional scaling ordinations of the bacterial community data**. Dotted lines are percent similarity contours. Abbreviations are as in Figure 3.

NMDS ordination of the bacterial community data revealed a pattern similar to that of the PLFA data, with the prevailing trend being segregation by location and a lack of trends associated with forest type or age within teak stands (Figure 6). CLUSTER analysis showed bacterial communities were at least 80% similar across all soils (Figure 6). Two-way nested ANOSIM revealed no significant differences among Teak soils, while two-way crossed ANOSIM showed that both forest type and location were significant (*p* = 0.058 and *p* = 0.006, respectively). CAP analysis using forest type as a factor gave strong canonical correlations (δ_1_ = 0.93, δ_2_ = 0.86) and by permutation yielded a highly significant $\delta_{1}^{2}$ of 0.789 (*p* = 0.004) and a highly significant trace statistic of 1.157 (*p* = 0.001). Ordination of the CAP results showed Teak soils clustering near the center of CAP1, while CAP2 separated samples by location with the north and south soils segregating to the bottom and top, respectively (Figure S5). Phyla that characterized the northern samples (Teak and native forest) were *Acidobacteria* and candidate phylum AD3 (Figure S5). For the south location, *Planctomycetes* characterized the Teak soils, while *Actinobacteria* and candidate phylum OP3 were more strongly correlated with the native forest.

**Figure 7:**
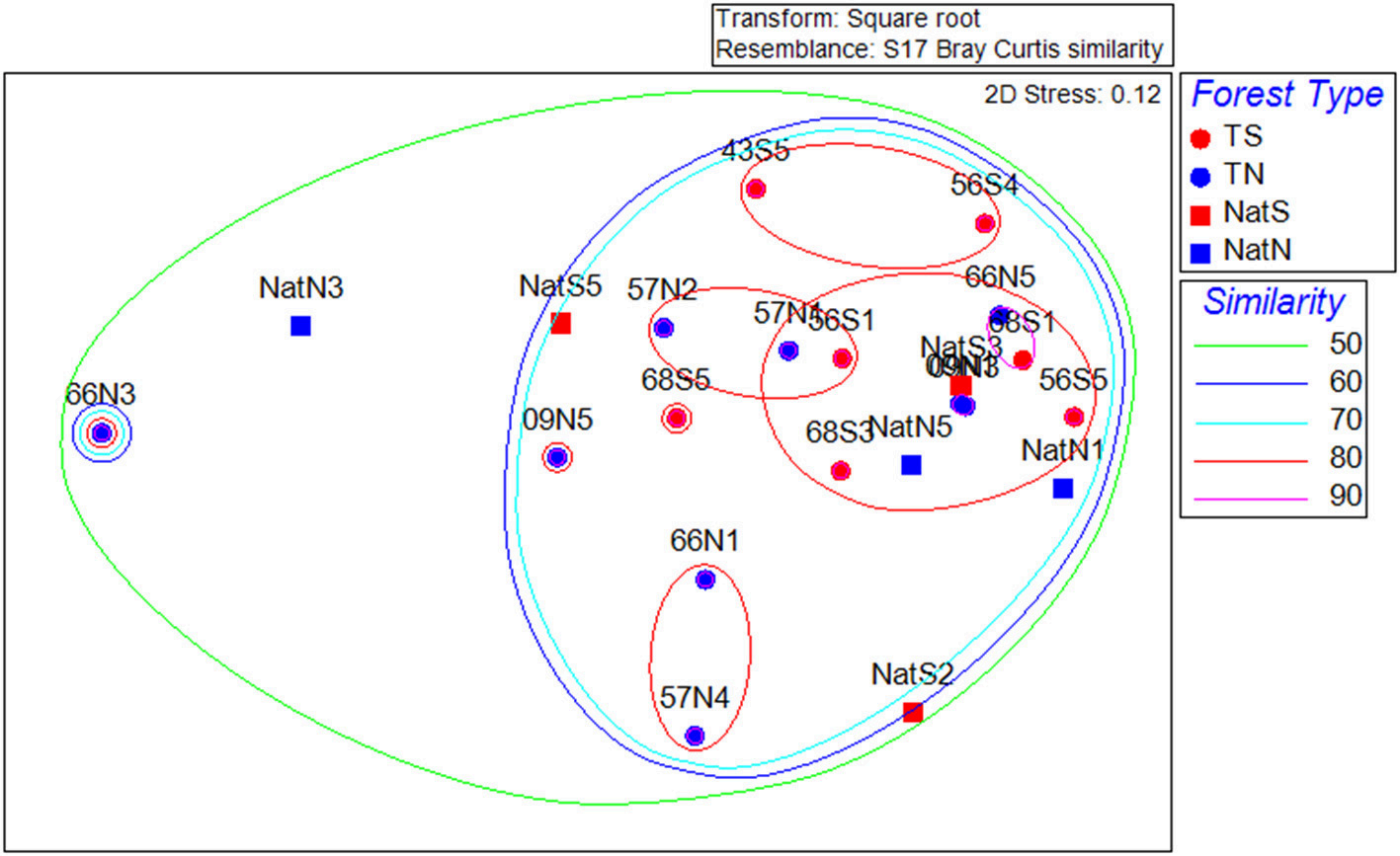
**Non-metric multidimensional scaling ordinations of the fungal community data**. Dotted lines are percent similarity contours. Abbreviations are as in Figure 3.

The NMDS ordination of the fungal community data was distinct from that of the PLFA data or of the bacterial communities, and showed no apparent trends in the data (Figure 7). CLUSTER analysis showed fungal communities were at least 60% similar across all soils. Two-way nested ANOSIM revealed no significant differences among Teak soils (*p* = 0.058), while two-way crossed ANOSIM showed that both forest type and location were significant (*p* = 0.006).

Potential correlations of bacterial and fungal community structure to edaphic properties were examined by using the RELATE and BEST tests. For the bacterial communities, RELATE gave a Rho value having relatively low strength and significance (0.224, Rho = 1 indicates a variation in soil properties exactly matches variation in community structure; *p* = 0.0588), whereas BEST analysis gave a no significant correlations. Fungal communities showed no correlation to soil properties: RELATE yielded a Rho of −0.206 (*p* = 0.938) and BEST analysis returned no significant correlations.

## Discussion

Soil enzyme activities were used as additional proxies of ecosystem functions (Bowker et al., 2011; Bailey et al., 2013). EAA serve as functional indicators and variation in production are often linked to changes in microbial community structure or activities, which are also impacted by resource inputs. The battery of EAA tested here examined processes in C, N, and P cycling. Detected differences in two: xylosidase (active on xylan and other related xylooligosaccarides), and acid phosphatase. But, effects were limited to the north location and there were no consistent trends associated with either forest type or Teak stand age. Thus, for EAA there was no evidence of significant impacts on functional activities of microbial communities associated with the conversion to Teak.

Microbial biomass changes in response to carbon inputs (Hopkins et al., 2014). But in the present study, conversion to Teak and associated shifts in organic matter inputs had no significant effect on overall amounts of microbial biomass. Variation in the composition of microbial biomass can also be used as a broad indicator in the abundance of specific guilds (total bacteria, GN-bacteria GP-bacteria *Actinomycetes*, and total fungi as well as AMF) and the ratios of guilds (GP:GN, F:B) (Smith et al., 2015). For all soils, GP:GN > 1 which indicated GP-dominated biomass, and on average the GP:GN ratio was higher in native forest than in Teak (but differences were not significant). The F:B ratios are widely used because of the major differences in metabolic processes of fungi and bacteria (Lundquist et al., 1999; Thiet et al., 2006). All soils the F:B < 1 indicating bacterial dominance of biomass. A key environmental property that the F:B often parallels is the C:N, with fungi favored by increasing C:N (Beare et al., 1992; Lundquist et al., 1999). In the present study, all soils had a relatively low C:N ranging from 7.2 to 12.8, and thus the low F:B may reflect the low C:N, which favors bacteria (Högberg et al., 2007; Lamarche et al., 2007; Fierer et al., 2009). However, it was also apparent that on average F:B in Teak stands was generally greater than that of the native forest, and that there was much greater variability in the F:B of Teak stands, with some F:B values approaching 2. Thus, although differences between Teak and native forest in GP:GN and F:B were not statistically significant, patterns in both of these ratios suggested the conversion to Teak plantation altered broad composition of the biomass in these soils.

Significant shifts in specific microbial guilds were detectable only at the south location, but these generally did not reflect trends as a function of forest type or of Teak stand age. The single exception was the significant decrease in the abundance of *Actinomycetes* in the Teak soils. *Actinomycetes* are notable in bacterial decomposition of plant polymers (De Boer et al., 1996; Romaní et al., 2006; Pandey et al., 2007). There is the possibility that *Actinomycetes* had a niche established to native forest since they were significantly less abundant in all Teak soils than in the soils of the native forest.

One of the questions in this study was whether a conversion from a diverse forest to a monoculture tree species would have impacts on broad diversity characteristics (species richness or evenness) and/or on specific aspects of community composition. This question was addressed by examining both bacterial and fungal community diversity *via* Illumina sequencing of 16S rRNA genes and ITS regions, respectively. The hypothesis was that conversion from diverse native forest to Teak would be reflected in decreased diversity in both bacterial and fungal communities and that alteration in diversity would be increasingly revealed with time. However, there were no significant differences in species (OTU) richness or evenness for any of the soils for either the bacterial or the fungal communities. In contrast, other studies showed shifts in bacterial and fungal diversity from four Alpine forest sites (Siles and Margesin, 2016), in a subtropical montane forest and bacterial diversity (Singh et al., 2014). The similarity between soils was also apparent in the fact that, for both the bacteria and fungi, identities of the OTU that dominated the respective communities was the same across all soils. Thus, conversion of the native forest to Teak plantation had no effect on the broad diversity characteristics of either bacteria or fungi in soils with forest ranging from 6 to 72 years of stand age.

While broad diversity parameters were not affected by conversion to Teak, changes in microbial community structure were detectable by univariate analyses of the abundance of specific constituents. For bacteria, this analysis showed that *Cyanobacteria* and *Nitrospirae* were negatively affected by conversion to Teak, while the candidate phyla TM7 and WS6 responded positively. The former two groups are involved in nitrogen fixation and oxidation of N oxides, respectively, and thus changes in these communities may have impacts on aspects of N cycling. The physiology of the candidate phyla is unknown, and thus potential impacts on ecosystem processes resulting from changes in these communities cannot be predicted. For fungi, univariate analysis revealed that an unidentified class of AMF was positively affected by conversion to Teak plantation. The AMF are of particular interest because Teak was colonized by AMF rather than by ectomycorrihzal fungi that are symbionts of the native forest tree species. Moreover, while more than 20 OTUs were assigned as AMF, only this one unidentified AMF class showed a significant correlation with the Teak, and may be important as it may reflect the impact of a non-native plant species on the AMF community in general, and may be of particular importance to the health and productivity of these Teak plantations.

Multivariate analysis of community structure provided further evidence of impacts of Teak on soil biology. For both the bacterial community data (phyla abundance in 16S rRNA amplicon libraries) and PLFA data, there were significant correlations with forest type as well as location, while fungal community structure (class abundance in ITS amplicon libraries) was not correlated with either. Multivariate analysis identified bacterial phyla that characterized soils, which included correlation of *Actinobacteria* with the south native forest. This finding was consistent with that from PLFA analyses, which showed an increased abundance of *Actinomycetes* (a constituent of the *Actinobacteria*). Thus, a shift in *Actinomycetes* was identified by the convergence of two orthologous techniques.

It has been suggested that one of the main pathways by which vegetation can affect the structure and function of soil microbial communities is *via* altering physicochemical characteristics of the soil (Augusto et al., 2002; Russell et al., 2007; Williams and Rice, 2007; Angel et al., 2010; Castro et al., 2010; Mueller et al., 2012; You et al., 2014) through species specific litter chemistry (Ushio et al., 2008; Strickland et al., 2009). Some studies have shown that some soil properties are highly correlated with soil microbial community composition (Ushio et al., 2008; Strickland and Rousk, 2010). Contrastingly, in the present study, correlations of microbiological properties to edaphic characteristics were relatively weak. Of the three community structure data sets (which reflect a comprehensive analysis), only the PLFA showed consistent correlations to edaphic properties, and BEST analyses revealed that N characteristics along with carbon content (You et al., 2014) and CEC played a central role in shaping microbial community structure. Collectively, these factors may indicate that microbial community structure as reflected in PLFA was affected by shifting nutritional characteristics of soils. Bacterial communities displayed only a relatively weak connection to soil characteristics by RELATE, and returned no significant correlations from the BEST analysis. The bacterial and PLFA data sets were therefore consistent to the extent that significant correlations to soil properties were identified by RELATE, and may reflect the fact that vast majority of PLFA extracted from soils are bacterial molecules (Nichols et al., 1986; Sundh et al., 2000; Ranneklev and Bååth, 2003; Schoug et al., 2008). The lack of trends associated with the fungi could have reflected, at least in part, a much greater level of variability between samples in abundance of fungal taxa. It was notable that the fungal RELATE Rho value was negative, indicting a high level of within group variability, which may have reflected a “patchy” distribution of different fungal classes in the soils examined.

The central question addressed by this study was how microbial communities respond to environmental perturbation in the form of an ecosystem conversion from a native forest to Teak monoculture, and involved two contrasting concepts in microbial ecology. One concept concerned the linkage of plant communities with those of soil microbes, such that characteristics of the former (composition, diversity *etc*.) affect the latter (De Deyn et al., 2008; Wardle et al., 2012; Jassey et al., 2013). Evidence from the present study supporting this linkage was the differing abundance of certain bacterial phyla as a function of forest type. For example, in the case of the Teak plantations, the *Actinobacteria* (*Actinomycetes*) and a particular class of AMF. The other concept concerned the resistance/resilience of microbial communities (Fanin and Bertrand, 2016) in which there is either no change as a result of environmental perturbation (resistance), or a temporary change before returning to the initial state (resilience). In the present study, resistance of the bacterial and fungal communities to ecosystem conversion was reflected in the absence of differences in the broad diversity characteristics (species evenness and richness). On the contrary, other studies showed shifts in bacterial and fungal diversity from four Alpine forest sites (Siles and Margesin, 2016), in a subtropical montane forest and bacterial diversity in Forest in South Korea (Singh et al., 2014). Resistance of the bacterial and fungal communities to the conversion from native forest to Teak was also displayed by the dominance of a common core of species (OTU) across all soils. Thus, the study provided support for both of the above-mentioned ecological principles, and showed that they are not necessarily mutually exclusive.

## Conclusion

Conversion of diverse native forest to Teak monoculture had no detectable effects on the species richness or evenness of bacterial or fungal communities in plantation stands ranging from 6 to 72 years old. However, while broad diversity characteristics were not affected, examination of specific taxa (phyla) showed that there were significant differences in bacterial community composition between the forest types, and that variation in community structure may have been related to differences in N chemistry in Teak *vs*. native forest soils. Distribution of fungal taxa was highly variable and revealed no significant pattern. Thus, the shift in forest characteristics affected microbial community characteristics, with effects more apparent in the bacteria than the fungi.

## Author contributions

Vd: Conception and design of study; Interpretation and analysis of data; contributed to write up of manuscript; some laboratory analyses; generation of figures and tables. WH: Conception and design of study; Interpretation and analysis of data; contributed to write up of manuscript. IB: Conception and design of study; Interpretation and analysis of data; contributed to write up of manuscript; some laboratory analyses. SD- Field Sampling and some laboratory analyses and assisted with study design. DD: Field Sampling and some laboratory analyses and assisted with study design. MB: Assisted with study design and revising it critically for important intellectual content. MW: Assisted with study design and revising it critically for important intellectual content; generation of maps.

## Funding

The University of the West Indies, St. Augustine Campus, Trinidad and Tobago, Campus Research and Publication Fund CRP. 3. Nov14.14 and O.N. Allen professorship in Soil Microbiology, University of Wisconsin Madison.

### Conflict of interest statement

The authors declare that the research was conducted in the absence of any commercial or financial relationships that could be construed as a potential conflict of interest.

## Supplementary material

Figure S1 **Map of Trinidad showing forest sample sites**. Letters indicate forest soils sample sites and are abbreviated as: N, North; S, South; Nat, Native. Numbers indicate year of establishment of forests.

Figure S2 **Canonical analysis of principal components (CAP) and ordination for separation of microbial community structure by location based on PLFA data**. T, Teak; S, South; N, North; Nat, Native Numbers following letters indicate replicate number.

Figure S3 **Rarefaction curves for prokaryotes amplicon libraries**. Letters indicate forest soils sample sites and are abbreviated as: N, North; S, South; Nat, Native. Numbers indicate year of establishment of forests.

Figure S4 **Rarefaction curve for fungal amplicon libraries**. Letters indicate forest soils sample sites and are abbreviated as: N, North; S, South; Nat, Native. Numbers indicate year of establishment of forests.

Figure S5 **Canonical analysis of principal components (CAP) and ordination illustrating Teak soils clustering near the center of CAP1, while CAP2 separated samples by location with the north and south soils segregating to the bottom and top, respectively**. Letters indicate forest soils sample sites followed by year of establishment and are abbreviated as: N, North; S, South; Nat, Native.

Table S1 **Elemental properties**.

Table S2 **Normalized abundance of fungal libraries across forest soils**.

Table S3 **Normalized abundance of bacterial libraries across forest soils**.
