# Supplementary figures and images for: Microbial Community Structure and Function of Soil Following Ecosystem Conversion from Native Forests to Teak Plantation Forests
Source: Front Microbiol. 2016 Dec 9;7:1976. doi: 10.3389/fmicb.2016.01976 (PMC5145857; doi:10.3389/fmicb.2016.01976)

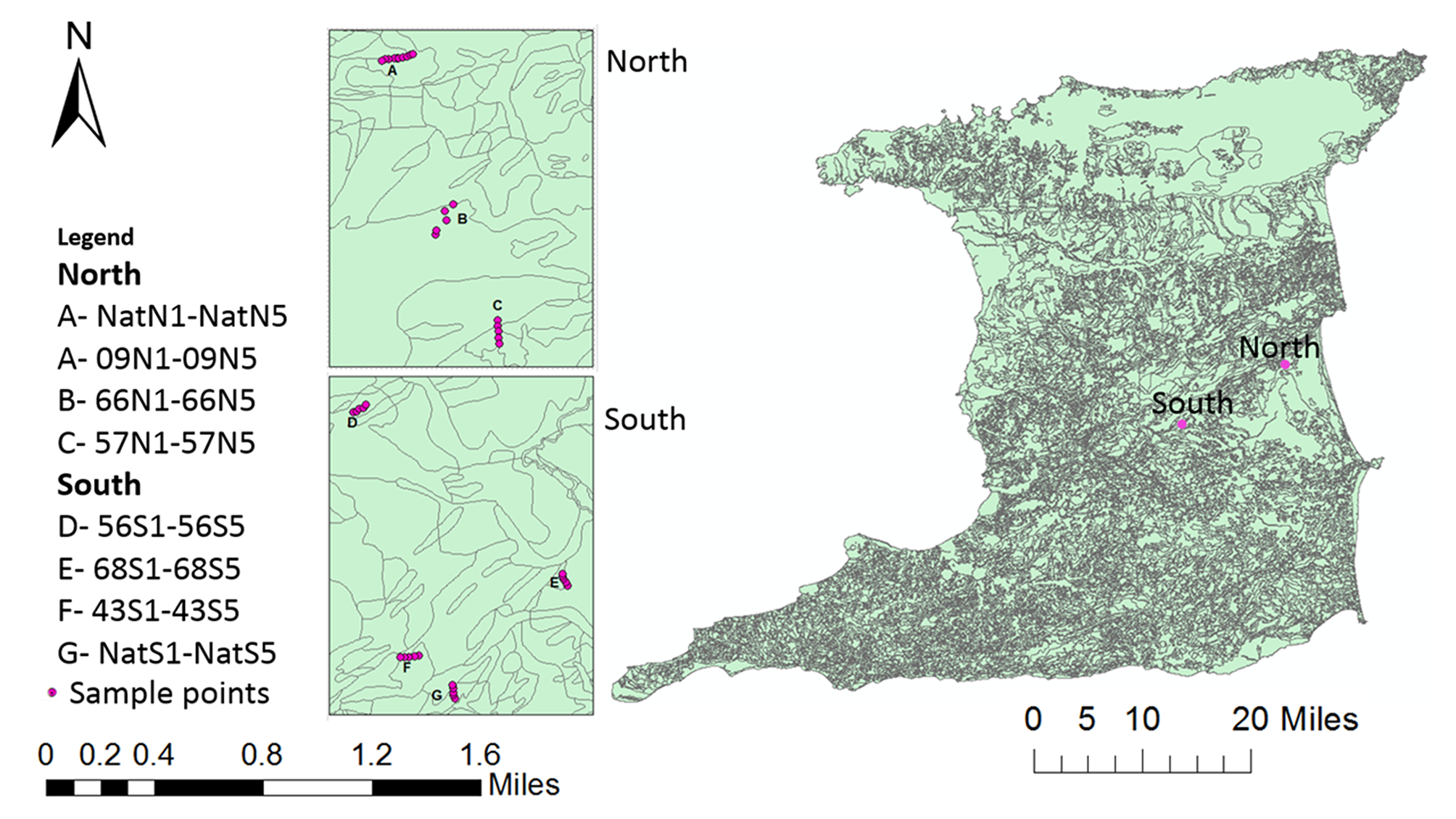

Supplement: Figure S1 — Map of Trinidad showing forest sample sites. Letters indicate forest soils sample sites and are abbreviated as: N, North; S, South; Nat, Native. Numbers indicate year of establishment of forests. [file Image1.TIF]

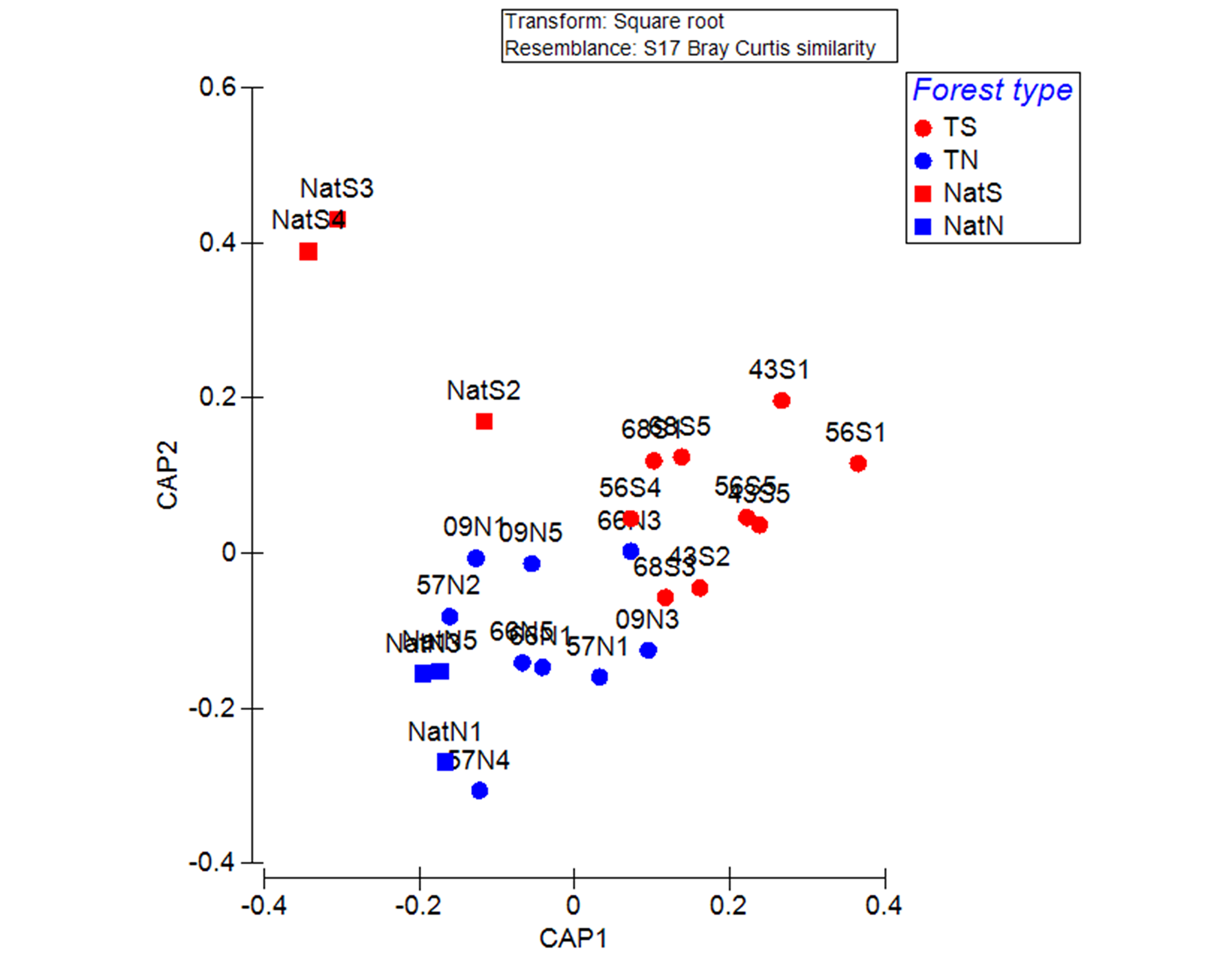

Supplement: Figure S2 — Canonical analysis of principal components (CAP) and ordination for separation of microbial community structure by location based on PLFA data. T, Teak; S, South; N, North; Nat, Native Numbers following letters indicate replicate number. [file Image2.TIF]

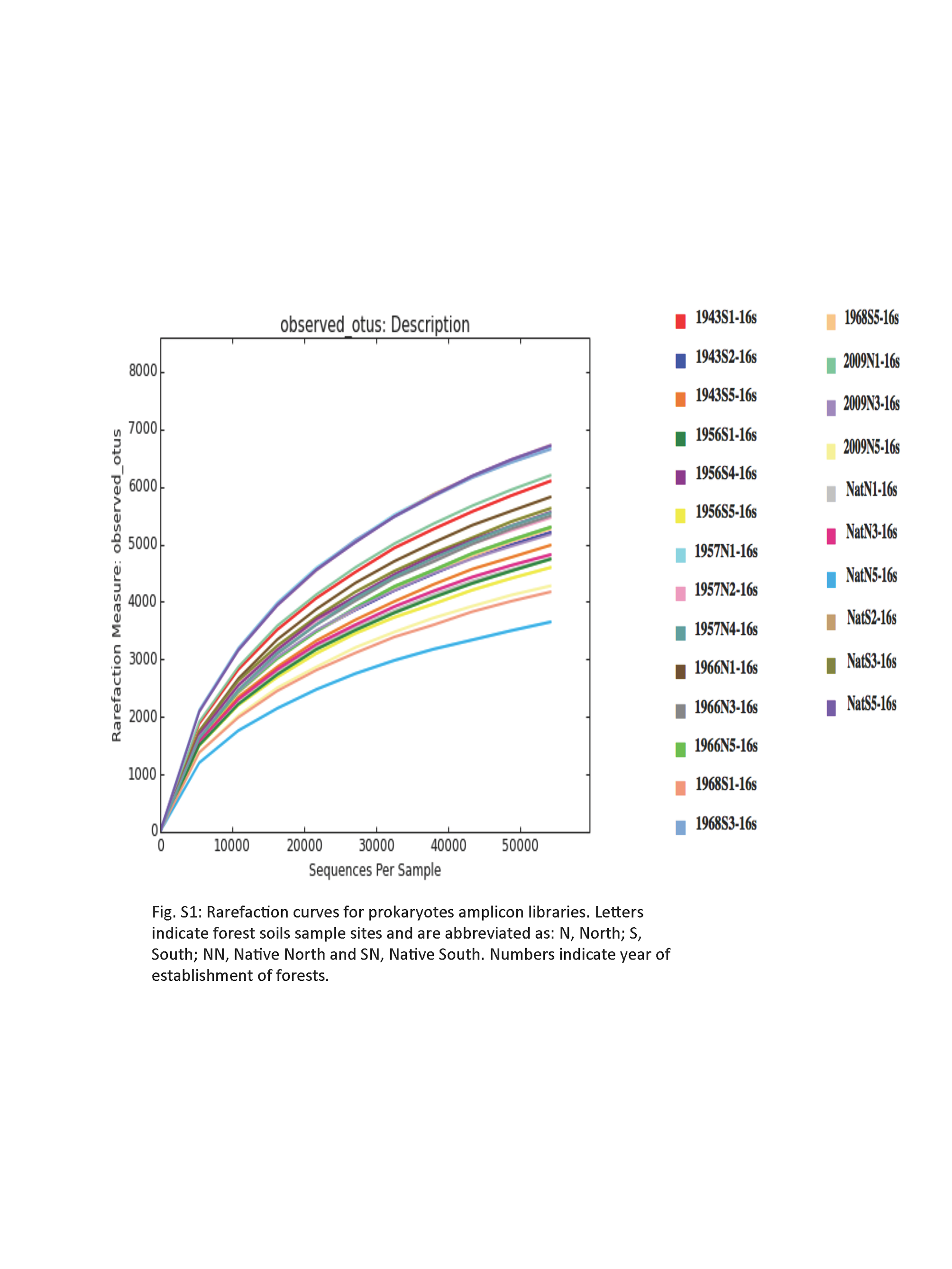

Supplement: Figure S3 — Rarefaction curves for prokaryotes amplicon libraries. Letters indicate forest soils sample sites and are abbreviated as: N, North; S, South; Nat, Native. Numbers indicate year of establishment of forests. [file Image3.TIF]

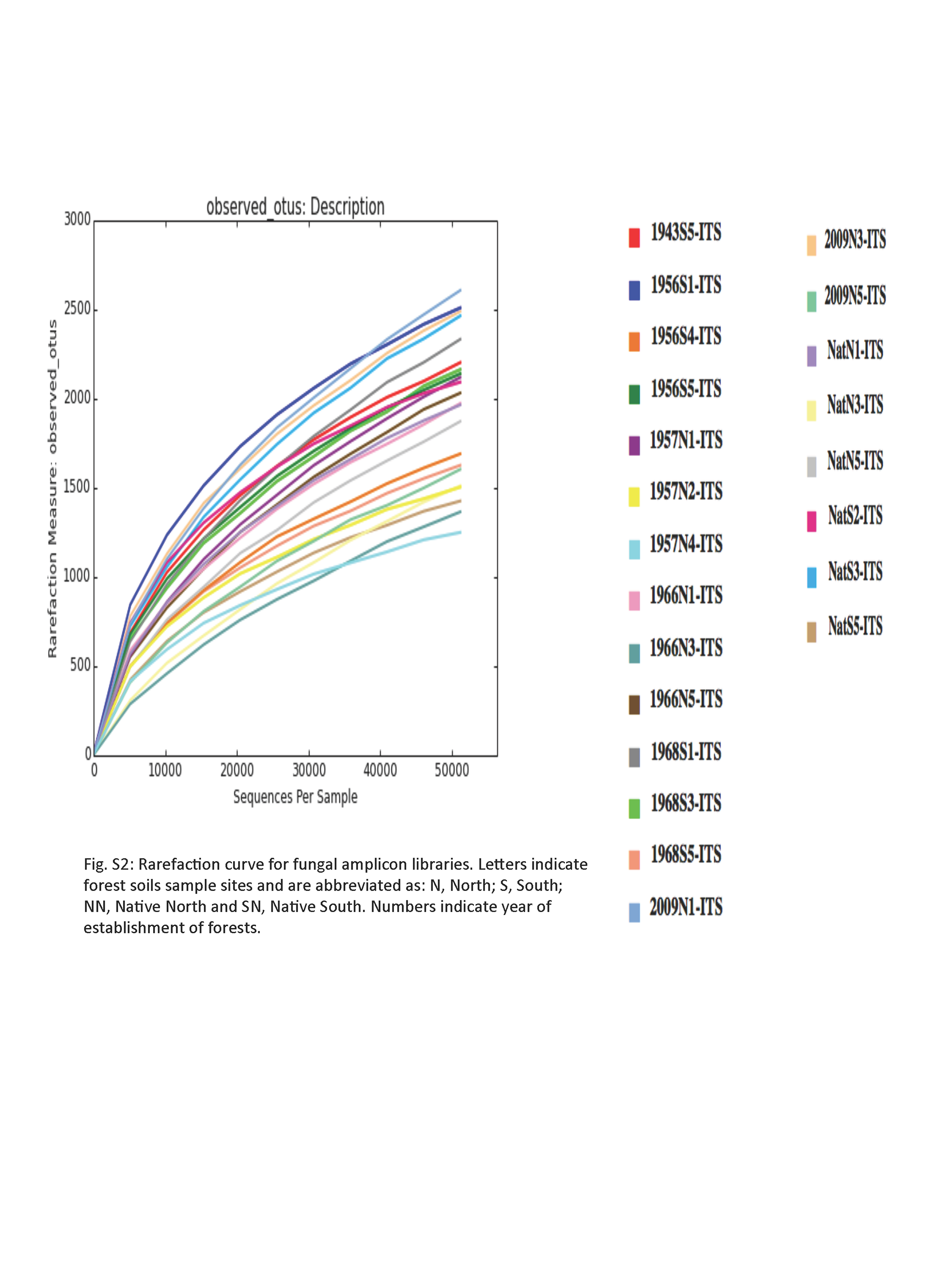

Supplement: Figure S4 — Rarefaction curve for fungal amplicon libraries. Letters indicate forest soils sample sites and are abbreviated as: N, North; S, South; Nat, Native. Numbers indicate year of establishment of forests. [file Image4.TIF]

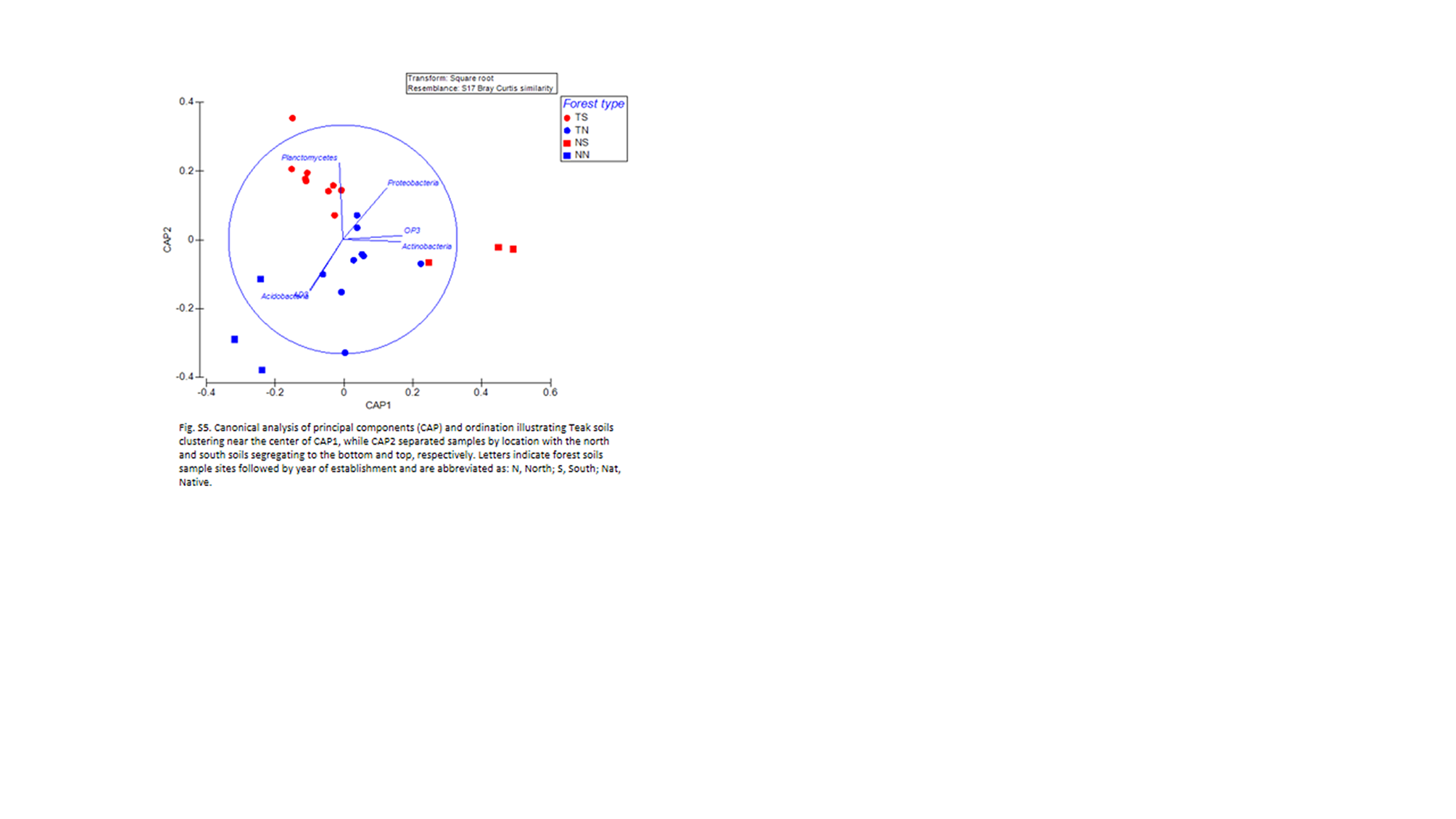

Supplement: Figure S5 — Canonical analysis of principal components (CAP) and ordination illustrating Teak soils clustering near the center of CAP1, while CAP2 separated samples by location with the north and south soils segregating to the bottom and top, respectively. Letters indicate forest soils sample sites followed by year of establishment and are abbreviated as: N, North; S, South; Nat, Native. [file Image5.TIF]
